# Advancing Dermatological Care: A Comprehensive Narrative Review of Tele-Dermatology and mHealth for Bridging Gaps and Expanding Opportunities beyond the COVID-19 Pandemic

**DOI:** 10.3390/healthcare11131911

**Published:** 2023-07-01

**Authors:** Daniele Giansanti

**Affiliations:** Centre Tisp, Istituto Superiore di Sanità, 00161 Rome, Italy; daniele.giansanti@iss.it; Tel.:+39-06-49902701

**Keywords:** telemedicine, mobile health (mHealth), remote monitoring digital solutions, dermatology, medical device, tele-dermatology, app, self-care, artificial intelligence, survey

## Abstract

Mobile health (mHealth) has recently had significant advances in tele-dermatology (TD) thanks to the developments following the COVID-19 pandemic. This topic is very important, as telemedicine and mHealth, when applied to dermatology, could improve both the quality of healthcare for citizens and the workflow in the *health domain*. The proposed study was centered on the last three years. We conducted an overview on the opportunities, the perspectives, and the problems involved in TD integration with mHealth. The methodology of the narrative review was based on: (I) a search of PubMed and Scopus and (II) an eligibility assessment, using properly proposed parameters. The outcome of the study showed that during the COVID-19 pandemic, TD integration with mHealth advanced rapidly. This integration enabled the monitoring of dermatological problems and facilitated remote specialist visits, reducing face-to-face interactions. AI and mobile apps have empowered citizens to take an active role in their healthcare. This differs from other imaging sectors where information exchange is limited to professionals. The opportunities for TD in mHealth include improving service quality, streamlining healthcare processes, reducing costs, and providing more accessible care. It can be applied to various conditions, such as (but not limited to) acne, vitiligo, psoriasis, and skin cancers. Integration with AI and augmented reality (AR), as well as the use of wearable sensors, are anticipated as future developments. However, integrating TD with mHealth also brings about problems and challenges related to regulations, ethics, cybersecurity, data privacy, and device management. Scholars and policymakers need to address these issues while involving citizens in the process.

## 1. Introduction

The use of telemedicine in dermatology is referred to as tele-dermatology. Historically, tele-dermatology (TD) has been arranged into two fields: the *real-time* (RT) TD and the *store-and-forward* (SaF) TD [1]. SaF TD allows a patient to contact a dermatologist through asynchronous consultations; this reduces the wait time for a meeting. *RT* TD is also frequently used in dermatology. It uses video channels to allow for interaction between the patient and the doctor.

Since 1995, there has been significant growth in the use of TD, with the number of publications reaching well over one thousand to date. A search on Pubmed, using the composite key in Box 1, *position 1* [2], highlighted 1287 papers on the date of this study, of which 590 (45.8%) were published starting 1 January 2020 during the years of COVID-19 pandemic. The use of TD has the potential to have an impact on the perceived quality of service delivery, improving the relationship between the actors in the *health domain* in terms of monitoring, treatment, and the process itself. This can lead to an increase in the satisfaction of both the patient [3] and of the other involved actors (e.g., general practitioners, specialists, assistants [4]). The combination of mobile health (mHealth) with TD can bring a synergistic benefit to the dermatology practice. TD, on the one hand, thanks to digital health solutions, can reduce travel distances or avoid travel entirely, as well as bring treatment directly to the patient’s home.

mHealth, on the other hand, by means of specific apps for smartphones or tablets, can place dermatological care directly in the hands of the citizen and of the other actors working in the field of dermatology. The real peculiarity of the integration of mHealth in TD, which is also present in dermatology, is the possibility of using apps directly by citizens to access virtual remote diagnoses and self-care in a decidedly stimulating way. This certainly opens the way for many opportunities, but also new problems. In other sectors of digital imaging, such as digital radiology and digital pathology [5,6], virtual interaction is, in fact, reserved exclusively for professionals in a working network connection environment. In addition to the opportunities and problems that have emerged in other digital imaging sectors, herein, we describe other opportunities and problems to be addressed. These considerations lead us to believe that among the challenges, perspectives, and problems involved in dermatology, the embedding of the mHealth into TD is especially significant [7,8].

The scientific literature in this field is available in Pubmed starting from 2012 (as it is possible to check after applying the composite key in Box 1, *position* 2 [9]). The interest in this topic is very recent, and has followed the mobile technology boom.

Box 1 **Composite keys used for the search in Pubmed in this section.**

           *(teledermatology [Title/Abstract]) OR (digital dermatology [Title/Abstract])*

       *((App [Title/Abstract]) OR (smartphone[Title/Abstract]) OR (mobile health[Title/Abstract]))*

          *AND ((Digital dermatology[Title/Abstract]) OR (teledermatology[Title/Abstract]))*

It was found that, on the date of this study, 87 studies had been published in total, of which 40 (45%) were published starting 1 January 2020 during the years of COVID-19 pandemic. The COVID-19 pandemic gave a further boost to biomedical technologies, including in this sector.

### 1.1. Key Pieces of Evidence before the COVID-19 Pandemic

The interest in this sector is documented by remarkable studies carried out in the months preceding the outbreak of the COVID-19 pandemic [10,11,12,13,14,15,16,17,18,19]. This is evidence of a maturation of this technology far beyond an embryonic state.

At the end of 2018, in a systematic review by Clark et al. [19], it was highlighted that with 4–6 billion people worldwide having access to mobile phones, TD could serve as a potentially useful tool for diagnosis and management. The authors reported a detailed overview of mobile phone technology and the accumulating evidence for its incorporation into dermatology. They addressed key questions, including regarding accuracy and concordance between mobile TD and face-to-face dermatology for the diagnosis of skin conditions.

Done et al. [18] focused on an interoperable informatics client-server system (VA Telederm) with a WEB server and apps accessing it as clients. VA Telederm worked as a provider server for web-based mobile apps designed to be integrated into the existing TD workflow of the US Veterans Health Administration (VHA). The results were strongly encouraging, showing that the use of mobile technology for consultative store-and-forward dermatology in a large healthcare organization was feasible. Koh et al. [17] hypothesized that mobile TD could facilitate skin self-examinations (SSEs) and further improve the monitoring and detection of melanoma. The study assessed the consumer acceptability and expectations of a mobile health app used to: (a) instruct SSE and (ii) conduct consumer-performed mobile teledermoscopy. The study reported that participants had positive views on using mobile TD to send images of skin lesions to a dermatologist or other medical practitioner. Silveira et al. [16] conducted a study in Brazil. The study indicated that the cell phone application (developed to aid in the diagnosis of skin cancer) showed great potential and reliability, and could, therefore, be considered as an ancillary option in countries with isolated communities that have limited access to dermatology clinics. Akdeniz et al. conducted a study [15] intended to stimulate primary health care professionals to play more active roles in the early diagnosis of skin cancer in nursing home residents. They concluded that, for the importance of the integration of mHealth with TD, dermoscopy courses, web-based or smartphone-based applications, and TD might support health care professionals in providing elderly nursing home residents with early diagnoses of skin cancer. Shambi et al. [14] investigated the features and overall quality of acne apps that could facilitate clinical management. They found that mobile acne apps could be effective in the self-management and collaborative management of acne. However, they also detected that the acne apps were of variable quality, and none contained all the features crucial for effective clinical management. Marwaha et al. [13] investigated the effectiveness and value of TD and face-to-face workflows for diagnosing lesions. They compared the risks of biopsy and cancer diagnosis among two face-to-face workflows (direct referral and roving dermatologist) and four teledermatology workflows. The results showed discrepancies, leading the authors to conclude that implementation with mHealth was critical to the effectiveness of TD. Damsin et al. [12] reported a synthesis of the objectives of the TeleSPOT project on the early detection of melanoma by TD in general practice. The TeleSPOT project used TD, a smartphone-based pigmented lesion diagnosis, and an online taskforce to provide a remote diagnostic aid for dermatologists to distinguish suspect pigmented skin lesions and accelerate their management. Tongde et al. [11] highlighted how mobile apps have become a part of the medical field, with dermatology being no exception. They reported that there were various types of dermatology apps, including *TD*, *self-surveillance*, *disease guide*, *reference*, *dermoscopy*, *conference*, *education*, *photograph storage and sharing*, *journal apps*, and others. The study examined the types of dermatology apps targeting patients and physicians that were most popular by analyzing their rankings in the Apple App Store.

### 1.2. The Unprecedented Impact of the COVID-19 Pandemic: The Idea of the Overview

TD in general and TD integrated into mHealth have undergone a very strong acceleration in interest in the last three years due to the pandemic. It is recognized that the COVID-19 pandemic has represented a real driving force for TD integrated with mHealth and artificial intelligence [10]. Generally, these developments led to the application of TD in order to carry out [10] remote diagnoses of problems relating to general skin pathologies and the monitoring and diagnosis of specific pathological problems with prognostic potential for the presence of COVID-19.

The passage of the image detection tools in the hands of the citizen has been completed due to the need to protect fragile subjects and to maintain social distancing. *But this does not mean that the transition process has been completed*, *given that the acceleration of necessity has also shown*, *inevitably*, *some critical issues*.

This has led to a paradigm shift in the citizen–patient and doctor–specialist relationships. This is a paradigm that we do not find in *teleradiology* or *telepathology*. All of this implies that the citizen becomes an operator/technologist, and this has exceptional implications that include various *aspects, such as, to name a few, digital divide, privacy and data security, technology, standardization, ethics, and specific training of use*. Furthermore, in addition to mobile technology, new technologies are appearing, such as artificial intelligence (AI). The latter, also integrated with the apps in the hands of the patients, has produced further important reflections by scholars, developers, and decision-makers in this area. In general, more domains of intervention are emerging in this area, ranging from technologies to ethical aspects.

Therefore, an overview focusing on the developments in the last three years in the integration of TD with *mHealth* can provide important added value to this field.

### 1.3. The Purpose of the Overview

The purpose of this narrative review proposal was to provide an overview of the current state of the field, considering the last three years of scientific production; to identify opportunities and challenges for integration; and to provide guidance for clinicians, researchers, and policymakers interested in advancing this area of healthcare.

The specific aims are as follows:

- To assess the trends and the evolution of the studies in this field;
- To assess the current state of the art in TD and mHealth, including their strengths and limitations;
- To identify the potential benefits of integrating TD with mHealth, such as increased accuracy and efficiency in diagnosing skin conditions, improved patient outcomes, and cost savings;
- To explore the challenges and barriers to integrating TD with mHealth, such as data privacy concerns, regulatory issues, and the need for specialized expertise;
- To provide guidance on the best practices for implementing and using TD and mHealth in dermatology, including recommendations for data collection and management, model development and validation, and clinical decision-making.

Overall, the topics addressed in the studies provide us with a useful idea of both the research directions and, indirectly, of the gaps and the bottlenecks to face.

## 2. Methods

The study was based on a *narrative review framework*. For the narrative review, after having defined the research question and sub-questions in the aims, we decided to follow: (a) a standardized checklist for narrative reviews and (b) a properly defined process of review. The process of review, reported in the algorithm below, necessitated the development of a search strategy. This included defining a process of selection, assessing the study quality, and conducting a data analysis identifying the emerging patterns following the selection process.

The *narrative review* used a standardized checklist designed for the category of narrative reviews (ANDJ checklist [20]) and a properly defined algorithm reported below (Algorithm 1).
**Algorithm 1 Algorithm applied in the overview**Set the search based on the composite key in Box 1 *position 2*:Conduct a targeted search on Pubmed and Scopus using the search query from step 1;Exclude conference papers from the search results;Exclude studies published before 2020;Select studies published in peer-reviewed journals that focus on the application of Apps/mobile health in teledermatology;Apply a qualification procedure based on an assessment of the parameters, as in [20];Include the preselected studies in the overview.

## 3. Results

All of the studies returned by the algorithm for the *quality assurance* passed the qualification step defined in the Algorithm 1.

The search provided 40 components [21,22,23,24,25,26,27,28,29,30,31,32,33,34,35,36,37,38,39,40,41,42,43,44,45,46,47,48,49,50,51,52,53,54,55,56,57,58,59,60] in the three years since the COVID-19 pandemic outbreak (at the date of this study).

In detail, the components were:

- Two randomized controlled (clinical) trials (RCTs) [37,49];
- Three observational studies (OS)s [39,46,47];
- Nine reviews [22,23,24,25,28,29,30,36,38] and one systematic review [32];
- Twenty-five papers [21,26,27,31,33,34,35,40,41,42,43,44,45,48,50,51,52,53,54,55,56,57,58,59,60].

### 3.1. Data Synthesis Plan

We decided to analyze the contributions separately. It was important to separately investigate the clinical randomized trials, observational studies, reviews, and papers on the application of digital dermatology in mHealth in this narrative review. This provides the following. (I) A comprehensive overview: By including different types of studies and critical evaluations, a narrative review can provide a comprehensive overview of the current state of knowledge on the integration of digital dermatology with mHealth. This can help readers to understand the different types of evidence that exist and how they contribute to the overall understanding of the topic. (II) Better ranges of evidence for analysis: Different types of studies have different levels of evidence, and it is important to evaluate the quality of the evidence of each type. For example, clinical randomized trials are considered the gold standard for evaluating the effectiveness of treatments, while observational studies are more useful for identifying potential correlations between variables. Scientific papers tend to be more focused on the specific research question, methodology, and data analysis, with a strict angle and focus on the research problem being investigated. On the other hand, reviews are broader, and consolidate fields by analyzing and synthesizing a large body of literature to identify patterns, trends, and gaps in knowledge. Reviews may cover multiple studies, experiments, or research areas and draw conclusions based on the analysis of the collective evidence. The authors of reviews may also critique the research methods and findings of individual studies, but their focus is on providing an overview of the field. By analyzing the evidence from different type of manuscripts, readers can understand the strengths and limitations of the existing evidence base. (III) Identifying gaps in the literature: Including different types of studies and critical evaluations can help to identify gaps in the literature and areas where further research is needed. For example, if there are too few clinical randomized trials on the application of digital dermatology in mHealth, this could indicate a need for further research in this area. (IV) A balanced view of the evidence: By including different types of studies and critical evaluations, a narrative review can provide a balanced view of the evidence. This can help readers to understand the complexity of the topic and avoid oversimplifications or overgeneralizations. For example, if there are conflicting findings between different types of studies, the review can present these findings and discuss possible explanations for the differences. Overall, separately investigating clinical randomized trials, observational studies, reviews, and papers in a narrative review on the application of digital dermatology in mHealth is important to provide a comprehensive, high-quality, and balanced overview of the existing evidence and to identify areas where further research is needed.

**Figure 1:**
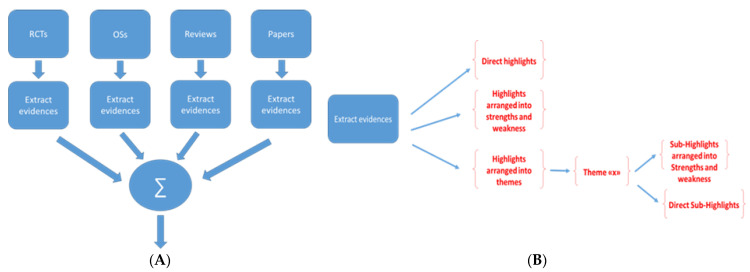
Data synthesis plan (**A**). Arrangement of the evidence based on the volume of production and the output (**B**).

Figure 1 shows the arrangement of the data synthesis. We proceeded by category of scientific study (Figure 1A). Based on the results of the studies and the number of studies in a category, we proceeded by reporting either direct evidence; evidence based on strengths and weaknesses (opportunities and bottlenecks, for example); or themes which then may follow, in an up–down process, an organization based on direct evidence or on strengths and weaknesses (Figure 1B).

### 3.2. Data Synthesis of the Overview of Scientific Papers

The analysis suggests a possible categorization according to the following emerging fields of interest detected in the scientific papers:

1. Opportunities of TD and mHealth. These studies have mostly focused on the horizons of application of TD and mHealth.
2. Development, implementation, testing, and shared methodologies. The studies that have addressed these topics have focused on the process of development, testing, and sharing of methodologies.
3. Integration models of TD and mHealth. These studies have addressed the issues of integration of TD and mHealth into specific models of the health domain.
4. The problems and bottlenecks. These studies have mainly addressed problems and bottlenecks of the integration of TD and mHealth into the health domain.

Some studies, in addition to having dealt primarily with one of the issues identified above, have also dealt with other issues which overlap in a secondary way.

#### 3.2.1. Opportunities of TD and mHealth

Generally, all the studies converged on the opportunities and general perspectives regarding TD in mHealth and its role in improving the quality of service overall [21,26,27,31,33,34,35,40,41,42,43,44,45,48,50,51,52,53,54,55,56,57,58,59,60]. The evidence showed that *TD and mHealth* could improve the quality of care, the healthcare process, the cost savings, the stress in healthcare facilities, and citizens’ satisfaction. However, some studies have focused more on bottlenecks, others on reviewing models of care, and still others on the specifics of the development and requirements, as described in the appropriate section.

Eight studies have specifically documented the opportunities in different sectors of dermatology.

Gandhi et al. [21] showed that a proposed TD application could improve the accuracy of the diagnosis of vitiligo, a challenging condition to diagnose clinically. The use of this app demonstrated the potential of TD to improve the diagnosis of skin conditions, including vitiligo.

Handa et al. [42] assessed the both the actors’ acceptance of and opinions on TD during the COVID-19 pandemic. They found *TD and mHealth* to be a valid alternative to in-person visits during the current crisis, with potential for even greater acceptability with further refinement of the process.

Hampton et al. [44] assessed, by means of interviews with citizens, the usability of an app proposed to guide their skin care. They found that the interviewed citizens considered the tool very user-friendly; however, they did not take sides on its usefulness in solving skin problems.

Yotsu et al. [45] aimed to evaluate the efficacy of an app (*eSkinHealth*) in improving the initial recognition and management of diseases at the skin level, including neglected tropical diseases, in sub-Saharan Africa through a mixed-methods pilot trial. The trial consisted of three phases, and the primary finding of the project was planned to be the volume of cases identified and followed by means of the app.

Tognetti et al. proposed a study [51] evaluating the actors’ approval and acceptance of the *MoleMe* App, a TD service based on AI for melanoma screening. They concluded that it had the potential to achieve widespread usage.

Peracca et al. [52] evaluated the frequency of use and the feasibility of mobile mHealth tools among dermatologists in a TD setting. They concluded that these tools allowed for adequate diagnostic accurateness to be reached.

The TELESPOT project in Belgium was reported in [57]. In this project, a smartphone-based dermoscopy application that could improve the diagnosis and treatment of skin cancer was developed. The project was successful in its user-friendliness, in its effectiveness, and in its trustworthiness, as demonstrated by means of specific feedback obtained in the interviews.

Sondermann et al. [59] highlighted that *TD and mHealth* were able to successfully reduce spatial and temporal barriers to dermatological care. Remote diagnosis was possible in 90.3% of the cases, and 64.3% were treated using only the TD.

Overall, the data synthesis highlighted several opportunities involving the integration of TD and digital dermatology with mHealth, including:

- Improving the accuracy of skin condition diagnoses, especially for challenging conditions like vitiligo, using TD apps;
- The potential for *TD and mHealth* to be a valid alternative to in-person visits during the COVID-19 pandemic; even greater acceptability will likely be reached with further refinement of the process;
- The use of mobile phone applications to assess photos taken by patients to improve the value, reliability, and availability of patients’ photos to guide their skin care;
- The potential for apps like eSkinHealth to increase the initial recognition and management of skin diseases, including neglected tropical diseases, in sub-Saharan Africa;
- The potential of AI-based TD services like MoleMe App for melanoma screening to achieve widespread usage;
- The feasibility of using mobile devices in TD settings to achieve adequate diagnostic accuracy;
- The development of smartphone-based dermoscopy applications in large national projects, like the TELESPOT project in Belgium, to improve the diagnosis and treatment of skin cancer;
- The successful reduction in spatial and temporal barriers to dermatological care through *TD and mHealth*, with remote diagnosis and treatment being possible in many cases.

#### 3.2.2. Development, Implementation, Testing, and Shared Methodologies

Five studies specifically focused on the design specifics, design requirements, and procedures in this field. Ritvi et al. [27] specifically described the development, implementation, and testing of a smartphone app in Norway that allowed referring physicians to send imaging cases of skin lesions to dermatologists for analysis and therapy. Use of the app helped patients to avoid referral to a specialist for regular consultations in 70% of cases, while 9% of patients were referred for systematic consulting with a dermatology specialist. The app helped patients to avoid many ordinary consultations for skin lesions with specialist healthcare services.

Abbott et al. [43] discussed the increasing use of mHealth in dermatology due to the COVID-19 pandemic, and proposed a guideline called CLOSE-UP as a shared methodology to guide clinicians in capturing and delivering high-quality clinical images.

Po Harvey Chin [50] focused on the development of a mHealth tool for the remote self-assessment of digital ulcers in patients with systemic sclerosis. The tool included an app, a custom color reference sticker, and a smartphone holder. The study aimed to evaluate image quality measures, the impact of automated feedback, and the feasibility of deploying the mHealth tool for home-based chronic wound self-monitoring by patients. The results suggested that the feedback mechanism improved the images overall.

Huang et al. [55,56] investigated the potential of an inexpensive device called NurugoTM Derma, focusing on its design characteristics. They reported the design and testing of the device. They also: (a) highlighted its potential as a tool for preliminary triage to detect those skin lesions requiring a face-to-face consultation with a specialist; And (b) reported the diagnostic validity of using images acquired by means of a NurugoTM smartphone microscope compared to a conventional dermatoscope for remotely diagnosing skin tumors or dermatological diseases through teledermoscopy.

Overall, based on the data synthesis, the studies reported their findings on the integration of TD into mHealth, including the following:

- A smartphone app was developed in Norway that allowed referring physicians to send information related to skin lesions to dermatologists for diagnosis and therapeutic advice, which helped patients to avoid referrals with specialists for regular consultations in 70% of cases, and could help them to avoid many ordinary consultations in specialized centers.
- Guidelines were developed based on a shared methodology called CLOSE-UP to guide clinicians in capturing and delivering high-quality clinical dermatological images through mHealth during the COVID-19 pandemic.
- An mHealth tool was developed for the remote self-assessment of digital ulcers in patients with systemic sclerosis, which included an app, a custom color reference sticker, and a smartphone holder, and showed that the feedback mechanism improved the overall image quality.
- The investigation of the potential of an inexpensive device called NurugoTM Derma for remotely diagnosing skin tumors or dermatological diseases through TD, which could be used as a triage tool to detect the cases requiring direct interaction with specialists.

#### 3.2.3. TD, mHealth, and the Integration Models

Six studies specifically focused on the models of *TD and mHealth* and on the relevant implications.

Trinh et al. [53] assessed the *organizational readiness* for change (ORC) for the purpose of implementing a patient-facing asynchronous mobile TD application, and suggested that the ORC survey may be a useful tool for identifying favorable areas for the implementation of TD through mHealth.

Veronese et al. [54] piloted a new model of care for *TD and mHealth* for elderly patients in senior living communities. This tool allowed for automatic, real-time self-identification of skin problems.

Kho et al. [41] explored different business models in *TD and mHealth*, and identified important partnerships, clinician involvement, management of biomedical ethical and legal risks, and responsibilities as key elements for the future of TD and mHealth services.

Yadav et al. [35] assessed patients’ satisfaction with a smartphone-based hybrid TD model of care during the COVID-19 pandemic. They found that, while most patients were satisfied with the service, some concerns were raised about the quality of care compared to direct consultation, as well as about the difficulty of obtaining medications.

Johnson et al. [26] identified the barriers and facilitators to using an mHealth model for monitoring low-risk skin lesions in elderly dermatology patients. The study proposed a framework highlighting the interconnections of various themes, at both the patient and provider levels. The study’s results suggested that addressing obstacles and enablers from both domains was essential in *TD and mHealth*.

Kling et al. [34] found that *TD and mHealth* increased the capacity for follow-up care in dermatology patients after discharge, but did not significantly improve the timeliness of care transitions.

The key points related to the models, based on the data synthesis, were:

- *TD and mHealth* are generally suitable for providing remote dermatological care to patients;
- Organizational readiness for change is important for identifying favorable areas in *TD and mHealth*;
- Models of dermatological care for elderly patients in senior living communities based on *TD and mHealth* are available;
- Different business models have been explored, and important partnerships, clinician involvement, management of medico-legal risks, and liabilities have been considered very important for the future of this field;
- Patient satisfaction with smartphone-based hybrid TD models of care during the COVID-19 pandemic was assessed, and while most patients were content, some concerns were raised about the quality of care compared to direct consultations;
- Barriers and facilitators to using mHealth models for monitoring low-risk skin lesions in the elderly were identified, and a conceptual framework involving various themes was proposed;
- *TD and mHealth* have been found to increase the capacity for follow-up care for dermatology patients after discharge, but may not significantly improve the timeliness of care transitions.

#### 3.2.4. Problems and Bottlenecks

Six studies specifically faced the problems and bottlenecks of *TD and mHealth*.

Lull et al. [31] assessed the quality of publicly available German apps for patients with psoriasis using the Mobile Application Rating Scale (MARS). The study concluded that involving patients in the development and evaluation of health-related apps is crucial, since the factors that make an app appealing to users may differ between healthcare professionals and patients.

Han et al. [48] focused on privacy and security concerns in the healthcare framework based on the Internet of Medical Things, particularly in TD. Their article proposed a zero-watermarking scheme based on federated learning to address privacy and security issues in *TD and mHealth*.

Vestergard et al. [58] highlighted that *TD and mHealth* have potential as a useful integrated tool for the initial recognition of skin cancer, but this should be used in conjunction with face-to-face consultations for accurate diagnosis and management planning. The accuracy was lower than that of in-person evaluations, but the sensitivity was similar.

Cronin et al. [60] reported that camera distance and angle can affect color accuracy in medical photography, which is important for consistent diagnosis and treatment. This could increase the variability in the appearance of photographed skin over time.

Sun et al. reported the outcome of a focus workgroup in this field [33,40], highlighting some problems and bottlenecks.

The study by Sun et al. [33] evaluated the existing state of applications of mHealth tools and assessed the impact of the tools’ features on image quality. The study found the following: it is important to consider which applications have standardized features allowing for image quality improvement; and very few applications embedded more than one acquisition technique feature. Another study by Sun et al. [40] comprehensively surveyed the image utilization features and technical characteristics found in publicly discoverable digital skin imaging applications. The study categorized 20 post-image acquisition features into 3 groups: metadata attachment, functional tools, and image processing. Furthermore, the authors found that fewer than 50% of the tools asked for informed consent during the interaction; and that the standards of technology were nearly always either not applied or not described regularly. The study recommended that gaps in the consent procedure, the information privacy, and the policies employed in the data usage be addressed.

In addition, the three studies [26,34,35] described in the previous section reported some limitations of models of *TD and mHealth* specifically.

From the data synthesis, the following problems can be highlighted in TD and mHealth:

- Quality of publicly available apps: The outcome suggests that involving patients in the development and evaluation of health-related apps is crucial, as the factors that make an app appealing to users may differ between healthcare professionals and patients;
- Diagnostic accuracy: The outcome highlighted that, while *TD and mHealth* have potential for the initial detection of skin cancer, the accuracy in diagnostic applications was not at the level of face-to-face evaluations, and should, thus, be used in conjunction with face-to-face consultations for accurate diagnosis and management planning;
- Variability in photographed skin appearance: The camera distance and angle can affect color accuracy in medical photography, which is important for consistent diagnosis and treatment. This could increase the variability in the appearance of photographed skin over time;
- Lack of cybersecurity (also including consent and data privacy) and data use policies.

In summary, the problems involved in *TD and mHealth* which emerged include issues with app quality, privacy and security concerns, diagnostic accuracy, variability in the appearance of photographed skin, and lack of a clear cybersecurity approach.

### 3.3. Data Synthesis from the Observational Studies

The search reported three observational studies (OS)s [39,46,47].

The first OS, by Mostafa and Hegazy [39], highlighted the potential of *TD and mHealth* as a solution for maintaining dermatological services during the COVID-19 pandemic. The observational study was conducted in Cairo, Egypt. It reported that using synchronous and asynchronous TD models showed an overall satisfaction and future use score of 91%, a usefulness score of 93.7%, interface and interaction quality scores of 85.9% and 87.0%, an ease of use and learnability score of 87.8%, and a reliability score of 86.7%. The study concluded that *TD and mHealth* are efficient methods of triaging and treating patients, and reduce the risk of COVID-19 exposure for physicians and patients in heavily populated countries. The authors recommended further efforts in legislation to implement physician compensation for tele-dermatology where this did not previously exist.

Dusendang et al. [46], in the second OS, compared four different *TD and mHealth* workflows for patients with rashes, and considered how they affected the utilization of dermatology services. The study found that the likelihood of a follow-up dermatology office visit within 90 days varied significantly depending on the technology and workflow used, but was not related to the medical center or primary care provider. Technologies and workflows that offered mobility through a smartphone with a high synchronicity of communication were associated with the standardized co-management of rashes.

The last OS, proposed by Gimeno-Vicente et al. [47], aimed to determine the basic characteristics of WhatsApp consultations, to quantify the time spent on them, and to assess the emotional impact on dermatologists. The study found that acute inflammatory conditions, usually requiring medication, accounted for 74.1% of the messages. Nearly one-third of the dermatologists reported that WhatsApp consultations had a negative emotional impact on them, and 82.3% stated that they would prefer not to receive these messages. Therefore, the authors suggest that WhatsApp consultations can be useful, but need to be moderated.

Overall, the key elements emerging from the OSs included:

- The potential of *TD and mHealth* as an integrated solution for maintaining dermatological services during the COVID-19 pandemic, with an overall satisfaction and future use score of 91%, a usefulness score of 93.7%, and high interface and interaction quality scores;
- The importance of introducing legislation to implement physician compensation for tele-dermatology where this did not previously exist;
- The significant variation in the likelihood of a follow-up dermatology office visit, depending on the technology and workflow used in the field of *TD and mHealth*;
- The prevalence of acute inflammatory conditions in WhatsApp consultations, which accounted for 74.1% of the messages, and the negative emotional impact on dermatologists; nearly a third reported negative emotions and 82.3% would prefer not to receive these messages.

### 3.4. Data Synthesis from Randomized Clinical Trials

The overview returned two randomized clinical trials (RCT)s [37,49].

Domogalla et al. [37] highlighted the use of a disease management smartphone app to improve the mental health of patients with psoriasis. The study found that the app was able to induce a significant reduction in HADS-Depression scores, although further research is needed to assess the app’s use frequency and its relationship with the patient outcomes.

Zhang et al. [49] focused on the development of an image-AI-based system called SkinTeller App to assess the severity of psoriasis. The study found that the model outperformed the average performance of 43 experienced dermatologists, with a 33.2% performance gain in the overall PASI score. The app has been used in multiple hospitals, and has been confirmed to be an excellent alternative for accurate assessment by dermatologists and chronic disease self-management in patients with psoriasis.

Considered together, these two studies showed that the TD integration with mHealth:

- Improved access to healthcare: *TD and mHealth* can improve access to healthcare services for patients who may face geographic, financial, or other barriers to in-person appointments;
- Increased efficiency: *TD and mHealth* can help to reduce wait times and streamline the triage and treatment processes;
- Better patient outcomes: TD and mHealth can lead to better patient outcomes, such as more accurate diagnoses and improved management of chronic skin conditions;
- Enhanced patient engagement: *TD and mHealth* can empower patients to take a more active role in their healthcare by providing them with tools and resources to better manage their skin health;
- Improved mental health: The use of disease management smartphone apps may improve the mental health of people with psoriasis.

### 3.5. Data Synthesis from the Reviews

The reviews covered various aspects of the integration of *TD and mHealth*, sometimes in the context of the COVID-19 [22,23,24,25,28,29,30,32,36,38].

Glines et al. [22] studied the impact of technology, particularly imaging technologies, on the field of dermatology. The article discussed the use of *TD and mHealth* to improve diagnostic accuracy and provide access to dermatologic evaluations for underserved communities and those in rural settings. It was suggested that incorporating digital dermatology into clinical practice required legal frameworks to be addressed and reimbursement policies to be updated in order to benefit patient care.

Petracca et al. [23] evaluated the implementation of a mobile TD app at three Department of Veterans Affairs sites using a properly designed approach. The study evaluated the organizational readiness for change (ORC) and identified enablers, obstacles, and factors affecting its implementation. The results showed a high readiness for change, with an ORC score of 4.2 out of 5.

The study by Marasca et al. [24] was a review of the scientific literature on the applications of *TD and mHealth* for inflammatory skin diseases. The study found that *TD and mHealth* have been demonstrated to increase access to resources in the health domain, improving the access to specific dermatological care for people living in remote areas.

Kevderiane et al. [25] reviewed and discussed the use of *TD and mHealth* in the care of allergic diseases of the skin. This was studied both in everyday life and in the context of COVID-19. The review discussed the of *TD and mHealth* applications and their practical benefits for clinical trials. The study highlighted a high level of patient satisfaction. However, the review also discussed some of the limitations and challenges of these technologies.

Havelin et al. [28] specifically reviewed the use of *TD and mHealth*, including AI, in managing psoriasis. The authors conducted literature searches and reviewed research publications linked to apps containing the keyword “psoriasis” to answer key questions relating to this field. In addition, they searched for apps dedicated to “psoriasis” on the analytic website www.appannie.com (accessed on 9 June 2023), and reviewed research publications linked to these apps.

Lee et al. [29] discussed how the COVID-19 pandemic affected dermatology and how TD became a popular alternative to in-person visits. Mobile TD, which allows patients to monitor and forward images of suspicious skin lesions to dermatologists for remote medical evaluation, was shown to represent a useful communication tool between medical practitioners and patients. AI technology was also used to assess clinical images for skin cancer. The authors concluded that there is a future for TD and mHealth in skin cancer detection, based on the incorporation of direct-to-consumer mobile dermoscopy with mole-scanning artificial intelligence.

Perrone et al. [30] discussed the various areas of telemedicine that have evolved during the COVID-19 pandemic, including teleradiology, telecardiology, and *TD and mHealth*. The latter has enabled the early identification of diseases through diagnoses of cutaneous signs. AI has also enabled the early diagnosis and monitoring of infections.

Greis et al. [36] highlighted the advantages of mHealth and AI in dermatology, particularly in African countries with limited medical care and long distances between patients and physicians. However, the challenge of ethnic variation needed to be addressed to improve the accuracy of automated algorithms. To achieve this, the authors concluded that there must be an increase in the quantity of available clinical data, which would require the active participation of local healthcare providers and the dermatological community.

Blum et al. [38] discussed the advantages and potential risks of using artificial intelligence in dermato-oncology, particularly for skin cancer diagnosis and treatment. The advantages included increased efficiency and the ability for medical professionals to focus on patients, while the potential risks included a lack of trust and misclassification of benign lesions. The work also mentioned that smartphone apps could be useful for disease-specific information, but they required clear guidelines and proper implementation.

Mbunge et al. proposed a systematic review [32] on the use of the TD and mHealth in South Africa during the COVID-19 pandemic. The researchers found that South Africa adopted various digital solutions based on mobile technology during the pandemic, including SMS services, messengers (for example, WhatsApp), mHealth apps, eHealth and telemedicine, AI, chatbots, and robotics. However, these technologies faced many obstacles, including managerial and financial barriers, legal and policy barriers, infrastructure and technology barriers, and cultural barriers. The authors recommended that energy be invested in community networks, especially in rural/remote areas, to modify mHealth policies, to develop sustainable strategies for the mobilization of resources, and to link accessible worldwide initiatives supporting this mobile technology.

The reviews considered herein identified the following opportunities and problems, in which AI was also considered an element:

Opportunities:

- *TD and mHealth* can provide access to specialized care and improve patient outcomes;
- *TD and mHealth* can increase access to healthcare resources, improving access to specialized centers for people living in remote areas;
- *TD and mHealth* can enable the early identification of patients through diagnoses of cutaneous signs;
- *mHealth* and AI can be useful in dermatology, particularly in African countries with limited medical care and long distances between patients and physicians;
- *TD and mHealth* can be useful for disease-specific information, screening, disease surveillance, medication compliance, and communication during pandemics;
- Incorporating digital dermatology into clinical practice can improve diagnostic accuracy and provide access to dermatological evaluations for underserved communities and those in rural settings.

Problems:

- The adoption and implementation of *TD and mHealth* technologies, including those integrated with AI, face technical issues, legal frameworks, and regulatory barriers;
- Incorporating digital dermatology into clinical practice requires legal frameworks to be addressed and reimbursement policies to be updated to benefit patient care;
- Technical issues can negatively affect the adoption of *TD and mHealth* technologies;
- The challenge of ethnic variation needs to be addressed in order to improve the accuracy of automated algorithms;
- *TD and mHealth* technologies face infrastructural and technological barriers, organization and financial barriers, policy and regulatory barriers, as well as cultural barriers.

## 4. Discussion

The topic investigated in the overview, relating to the integration of Digital Dermatology and TD with mHealth, is particularly complex. In fact, the two technologies have historically shown important complexities in their integration with the *health domain*. The *first*, TD, inherits the problems of *telemedicine and e-Health*. These problems, such as their regulation, was only partially and patchily overcome during the COVID-19 pandemic [61,62]. The *second*, *mHealth*, has a very short history in terms of its connection to and integration with digital health imaging. It is based on apps installed on smartphones, and provides great opportunities to reach the entire population, but it also has its own problems.

### 4.1. Added Value

The integration of mHealth with digital dermatology and TD has accelerated rapidly during the COVID-19 pandemic, providing new opportunities for citizens and for all of the players in the *health domain*. This review intended to contribute to this by investigating the state of integration of this field with the *health domain* and to mark a point on the map. Our study was a narrative review. Narrative reviews are particularly useful in relatively young sectors for the process of medical knowledge construction. Systematic reviews are an important tool, but sometimes they are ineffective for application in very young scientific fields where there is still little medical knowledge. In this narrative review, after having defined the research question and sub-questions in the aims section, we decided to follow both a standardized checklist for narrative reviews and a properly defined review process. We performed an overview aiming to add scientific value to the field by acting as a connector between all the specific issues addressed in this field to date.

### 4.2. Interpretation of Results

#### 4.2.1. Opportunities

The overview showed various opportunities in this area, as assessed from studies that showed the acceleration of the development of this field during the COVID-19 pandemic [10] due to a state of necessity. However, we also found evidence of the maturation of this technology even before the pandemic, which progressed far beyond an embryonic state [11,12,13,14,15,16,17,18,19]. It was highlighted [19] that, at the end of 2018, with 4–6 billion of people worldwide having access to mobile phones, TD could serve as a potentially useful tool for diagnosis and management when integrated with mHealth. With the advent of mHealth-based solutions in TD, dermatology is witnessing a new paradigm of image diagnostics that has never been observed in other sectors, such as radiology and digital pathology. This paradigm consists of placing the diagnostic device directly into the hands of the citizen, who becomes an actor in the treatment process. In this way, citizens become able to use these devices themselves for many activities related to dermatology. Several categories of apps for citizen use have been identified in the dermatology field, including *TD, self-surveillance, disease guide, reference, dermoscopy, conference, education, photograph storage and sharing, and journal apps* [11]. The ability to carry out self-diagnosis in the dermatological field certainly represents an important technological advance in the health domain. Generally, all of the studies converged in terms of the opportunities and general perspectives of TD and mHealth in terms of improving the quality of services overall. Specifically, the studies showed that TD integrated with mHealth, as well as with AI, could improve the quality of care; healthcare processes; cost savings; the stress in healthcare facilities; citizens’ satisfaction; and general care, even in disadvantaged environments (as shown, for example, by a study conducted in Africa [26]). These opportunities cover many fields of application, including (but not limited to) the monitoring of acne, vitiligo, psoriasis, and skin cancers [12,14,17,21,28,51]. There have now been countless apps developed in this area, which are subject to analysis and revisions [11,19,28]. Self-diagnosis has been shown to be particularly useful and feasible in three important phases of the care process: *remote diagnosis* [17]; *triage* [55,56]; and after *discharge, at follow-up* [34]. It is applicable to *young people* (such as for monitoring acne [14]); *frail people* (such as for monitoring digit ulcers in patients with systemic sclerosis [50]); the *elderly* [26,54]; and *people living in disadvantaged areas* (where it has also proved to be useful for the evaluation of tropical skin diseases [45]).

The availability of important client (mobile device) and server (provider) architecture for storing images [18] also makes it possible to improve the training processes for professionals in the dermatological field [63,64]. As in digital pathology, which uses an electronic slide that can be accessed from a smartphone remotely, in digital dermatology, smartphone access to an archive of digital images can simplify a student’s training and free up laboratories for more important activities [63,64,65,66].

#### 4.2.2. Challenges and Barriers

The overview also showed that there are still many challenges and barriers facing this sector. If, on the one hand, bringing mobile tools into the hands of citizens has increased citizens’ self-awareness with respect to their dermatological health, on the other hand, it has highlighted that there are still numerous open questions and problems to be overcome.

First of all, it is true that mobile devices are experiencing a huge diffusion worldwide, but it is also true that the problem of the digital divide persists [67,68,69]. This issue has two polarities [67,68]. The first concerns infrastructures and devices [67], and affects more disadvantaged countries and territories in practice—precisely those who could greatly benefit from remote connections of both the client (mobile device) and server (care provider) types [16]. The second concerns so-called digital literacy [68], which has a greater impact on the elderly population [69], who could gain great benefits from these tools precisely due to the risk of many skin pathologies, which grows along with age [15,54].

This centrality of the citizen in the process of connection to the care service makes him a de facto citizen operator/technologist. This has a great impact on app- and hardware-based smartphone tools, which must be maneuvered to connect with healthcare providers; to send photographic documentation; and to interact with a complex, distributed, and interoperable system [38]. This implies that attention must be paid to various aspects, such as, to name a few, cybersecurity (including privacy and data security), the standardization of tools, technological innovation, and ethics [10].

Furthermore, in addition to mobile technology, new technologies are appearing, such as artificial intelligence (AI). The latter, which can also be integrated with apps in the hands of the patients, has determined further important reflections by scholars, developers, and decision-makers in this area.

In general, more domains of intervention are emerging in this area, ranging from technologies to ethical aspects.

Sun et al. [33,40], for example, proposed a study divided into two parts which addressed the critical domain of standardization from a global point of view. The take-home messages were that the image post-processing capabilities vary greatly based on the us-er and the intended function; technological standards were not always implemented or reported; and critical issues were also detected in the consent procedure and in other aspects of privacy and data confidentiality which were either not present or not standardized.

Blum et al. [38] remarked that both the advantages and the problems of TD in mHealth were also present when integrated with AI. They detected seven domains of interventions: (1) the lack of mutual trust generated by the diminished patient–physician direct relationship; (2) the additional time needed to assess the benign lesions; (3) the absence of sufficient medical knowledge to detect incorrectly classified AI decisions; (4) the need to contact the patient again rapidly in the case of incorrect AI classification; (5) medico-legal issues due to lack of regulation in this field; (6) the problem of reimbursement; and (7) the fact that apps based on AI are currently unable to provide adequate assistance based on an image of a positive test for a cancer skin lesion.

Tognetti et al. [51] identified three limitations, namely, -the current lack of a common and clear European regulation; the need to design efficient workflows and plans for better interaction among different actors; the absence of agreement initiatives, with particular reference to position statements and guidelines.

By overturning this point of view and focusing on the decision-maker/diagnostician, the overview demonstrated that all the difficulties on the patient’s side (which are wide-ranging) are reflected on the figure with the task of making the diagnosis (for example, the incorrect capturing of images). The lack of guidelines, specific legislation, clear rules on reimbursement procedures, and a clear definition of the workflow do not facilitate the work of this figure, who has a key role in the diagnosis and assumes maximum responsibility [51].

Looking at the entire process, this overview seems to converge on the finding highlighted by Blum et al. [38]. They reported the requirements for actors’ satisfaction in an mHealth process in the health domain, and stated that apps can be applied correctly if the quality of the images is adequate; if the patient’s data can be uploaded easily; if the data exchange allows for both the uploading of images and the downloading of results; and if the medical reimbursement and medico-legal aspects have been disclosed.

#### 4.2.3. Future Directions

As seen in the overview, an important contribution, both in TD in general and in its integration with mHealth, has been provided by artificial intelligence [10,29]. With this integration, we are witnessing an important trajectory of the future development as it is outlined. The important issues to be addressed along this evolutionary path have many aspects in common with other imaging disciplines, such as digital pathology and digital radiology [70], and these range from regulatory to ethical aspects. However, the fact that these devices are also managed by the *citizen–patient–operator* means that they require greater attention in this process of integration [71]. As for radiology and digital pathology [70], it will be very important to train AI-based algorithms to avoid population bias, both of the *intra-ethnic* and *extra-ethnic* varieties [36]. As highlighted in the overview, in relation to the study by Greis et al. [36], in order to improve the efficacy and usability of AI, there must be an increase in the amount of available clinical data. This requires the active participation of both local healthcare providers and the dermatological community.

**Figure 2:**
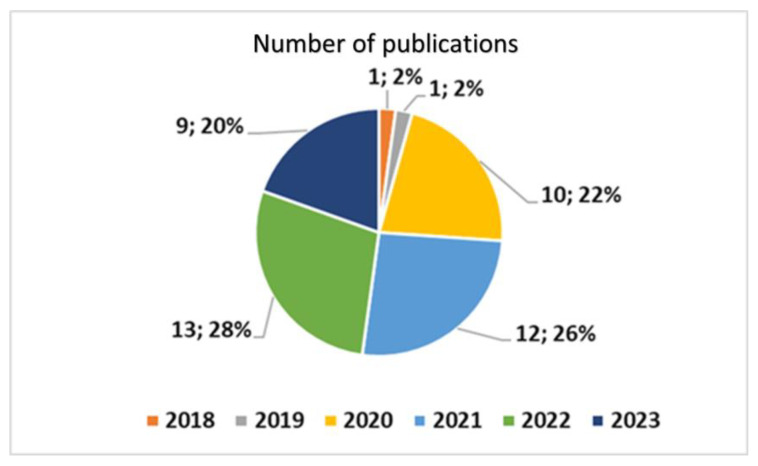
Yearly distribution of publications dealing with artificial intelligence and TD.

As can be seen in Figure 2, by applying the composite key in Box 2, *position 1*, to a search of Pubmed, it was found that the developments and publications (40 papers in total) in this field have decidedly been very recent, with 96% of publications published from 2020 onwards.

Another sector in which promising development is being witnessed is that of augmented reality (AR).

A Pubmed search using the composite key in Box 2, *position 2*, highlighted eight papers from 2019 (of which seven were considered to be relevant) [72,73,74,75,76,77,78].

Promising results were obtained by different dermatological studies regarding the feasibility, usability, and acceptability of using both Google Glass and Microsoft HoloLens in patient-centric settings, as well as in medical education and training [72]. Further work and development of rigorous research designs are required in order to evaluate the efficacy and cost-effectiveness of wearable AR devices in the future.

Interesting perspectives have been found in the cosmetological industry [73], where AR was evaluated as an appropriate means to respond to changes in the market when integrated with AI [76]. Virtual, augmented, and mixed reality are considered to play an important role in the education of preclinical, medical, and nursing university students, as well as in dermatology courses [74]. Learning outcomes such as student satisfaction, self-efficacy, and engagement have all been shown to increase along with the use of immersive technology, suggesting that it is an optimal tool for education [74].

In [75], the authors reviewed the benefits, limitations, and future directions of AR in relation to the measurement of dermatological conditions. This may include simple linear dimensions, area calculations, and even assessments that drive clinical interventions. The authors also highlighted the current baseline mobile application tools that may lay the groundwork for the further validation, augmentation, and utility of these technologies.

Based on the study reported in [77], virtual reality (VR) and AR have been making headlines, pushing the boundaries of educational experiences and demonstrating their applicability in a variety of fields. The authors briefly presented the status of VR/AR with regard to dermatology. In [78], the authors defined clinical and educational uses for AR in dermatology, and discussed key policy considerations for the safe and appropriate use of this emerging technology.

Wearable devices in general can represent an interesting integration resource for the future in this area. A search on Pubmed using the composite key in Box 2, position 3, highlighted only one review work [79], which was dedicated to Google Glass [79], the AR device that was treated in [72], among other things. By conducting less stringent searches, some studies were found that addressed this issue, several of which referred to the AR already discussed above. This indicated that, according to some authors, AR tools are considered to be wearable devices.

In [80], the authors reviewed the materials, design strategies, and powering systems used in soft electronics to providing an overview of the applications of these devices in cardiology, dermatology, electrophysiology, and sweat diagnostics, with an emphasis on how these systems may replace conventional clinical tools. A study on wireless soft sensors of skin hydration, with designs optimized for rapid, accurate diagnostics in dermatology, was presented in [81]. The authors of [82] highlighted how the rapid evolution of technology, sensors, and personal digital devices offered an opportunity to acquire health-related data seamlessly, unobtrusively, and in real time. They discussed the relevance and opportunities of using digital sensing in dermatology, taking eczema as an example. Wearable sensors have also been proposed to specifically quantify pruritic behaviors in dogs [83], but future perspectives for humans have also been suggested.

An important sector that has shown development in the past in dermatology is that of wearable devices for contact thermography [84]. Thanks to technological developments through algorithms and miniaturization [85], this sector could have important prospects for dermatology, and could be revived for future developments in telemedicine [86].

Overall, mHealth, AI, AR, and wearable devices are involved in the design of an attractive development scenario in the field of TD and digital dermatology. In this scenario, the *citizen–patient–operator*, who has an increasing role as an actor in the monitoring and treatment processes, will be able to connect with aspects of virtual healthcare through these tools, which will also be integrated with each other. However, it is important that scholars, doctors, healthcare professionals, and politicians address the important issues that will surround the process of integration into the health domain by tackling all of the intervention domains synergistically.

Box 2 **Composite keys used for the search of Pubmed.**

       *(teledermatology [Title/Abstract]) AND (Artificial Intelligence [Title/Abstract])*

        *(dermatology [Title/Abstract]) AND (Augmented Reality [Title/Abstract])*

         *(dermatology [Title/Abstract]) AND (wearable device [Title/Abstract])*

### 4.3. Limitations

This narrative overview has limitations. Our review considered papers written in English, along with two in German (available in Pubmed). Reviews in different languages which were not available in these databases were not considered. The PubMed and Scopus databases were consulted, and only peer-reviewed papers were considered in the review. Databases at the local/national level were not consulted.

## 5. Conclusions

The overview highlighted an acceleration of TD development in mHealth during the COVID-19 pandemic. During this period, telemedicine has allowed for the monitoring of dermatological problems with prognostic probabilities of viral infection, as well as remote specialist visits for citizens, in order to minimize face-to-face interactions. Both AI and smartphone apps provided important contributions in this area, as they are used directly in the hands of the citizen, who has become an *actor–operator–technologist* at once with these developments. The relationship between the patient and the tool represents a new paradigm compared to other imaging sectors, such as digital radiology and digital pathology, where the exchange of information is reserved for professionals. In these innovations, there are enormous opportunities, but also challenges and problems to overcome. Generally, all the studies converged on *the opportunities and general perspectives* of TD in mHealth. These aspects involved improving the overall quality of service, the healthcare processes, and the cost savings; minimizing the stress in healthcare facilities; increasing citizens’ satisfaction; and bringing a more punctual standard of care to those living in disadvantaged areas. These opportunities cover many fields of application, including (but not limited to) the monitoring of acne, vitiligo, psoriasis, and skin cancers. There are many prospects for future developments in addition to integration with AI. Integration with AR is also gaining ground in various sectors, such as the cosmetology sector, and integration with wearable sensors is being assessed for monitoring parameters of dermatological interest. The future direction of this research is to attempt to allow the patient to integrate different tools for dermatological monitoring.

However, the integration of TD with mHealth has also begun a very delicate process which has brought diagnostic devices, also equipped with artificial intelligence, into the hands of citizens. This adds to the long-standing regulatory, ethical, and medical–legislative problems (including reimbursement and consent) of telemedicine, which are still present here, along with the further problems of cybersecurity, data privacy, and greater attention to the development and management of devices in the hands of citizens (which will be at the heart of the process) in employment models (including hybrid ones). Initiatives are strongly requested by the scholars and politicians who intervene in all the necessary domains to also involve the citizens themselves in the conception, development, and release processes of these tools.
